# Network analysis in adolescent patients with somatic symptom disorders

**DOI:** 10.1186/s12888-025-07686-3

**Published:** 2025-12-27

**Authors:** Doudou Zheng, Shijian Wang, Jingya Li, Linghua Kong, Lin Zhao, Ying Yang

**Affiliations:** 1 Shandong Mental Health Center, Shandong University, Jinan, Shandong 250014 China; 2 School of Nursing and Rehabilitation, Cheeloo College of Medicine, Shandong University, Jinan, Shandong 250012 China

**Keywords:** Somatic symptoms disorders, Adolescence, Network analysis, Bayesian analysis

## Abstract

**Background:**

Somatic symptom disorder (SSD) is one of the most common conditions in adolescents. Persistent symptoms are closely associated with impaired academic performance, reduced social functioning, high healthcare utilization, and increased risk of comorbid psychiatric conditions such as depression, eating disorders, and self-harm. Despite its substantial burden, the clinical mechanisms underlying SSD remain poorly understood, limiting the development of effective, targeted interventions. Clarifying the interrelationships among core somatic symptoms may therefore provide critical insights for clinical management and improve treatment precision.

**Methods:**

A total of 585 patients were recruited between June 2024 and January 2025. All participants completed the Chinese versions of the Patient Health Questionnaire-15 (PHQ-15) and the Somatic Symptom Disorder-B Criteria Scale-12 (SSD-12). To quantify the structure of the somatic symptom network, centrality indices including strength and expected influence were used to identify key symptoms. Bayesian network analysis was further applied to explore potential causal relationships, offering the advantage of directional interpretation within the network framework.

**Results:**

The symptoms of nausea, dizziness, shortness of breath, and chest pain exhibited the highest centrality values, indicating that they occupied the most central positions within the somatic symptom network. In the Directed Acyclic Graph (DAG), “cannot breathe” is located at the top of the network, indicating its potential directional influences and being more likely to trigger other symptoms.

**Conclusion:**

While the evidence remains inconclusive regarding their direct suitability as treatment targets, psychological and neurobiological research may provide further insights into these central symptoms. Focusing on these nodes could inform future investigations and guide the development of more refined clinical strategies.

**Clinical trial number:**

Not applicable.

**Supplementary Information:**

The online version contains supplementary material available at 10.1186/s12888-025-07686-3.

## Introduction

 Somatic symptom disorder (SSD), introduced in the Diagnostic and Statistical Manual of Mental Disorders-Fifth Edition (DSM-5) in 2013, is characterized by distressing somatic symptoms (e.g., dizziness, palpitations, chronic pain, or gastrointestinal discomfort) accompanied by excessive thoughts, feelings, or behaviors related to these symptoms, irrespective of whether an identifiable medical condition is present [1–3]. Somatic symptoms are some of the most common complaints in adolescents [4]. The estimated prevalence of somatic symptoms among youths is between 25% and 75%, yet only 5%-7% of children meet the full criteria for SSD [5, 6]. Although most symptoms demonstrate a transient nature without meeting diagnostic criteria for psychiatric disorders or causing enduring functional limitations in daily life and developmental progression, persistent symptomatology in a subset of children and adolescents may lead to clinically significant impairment requiring intervention [7]. These physical discomfort symptoms are closely tied to excessive worry and maladaptive cognitive-behavioral responses, which often manifest as heightened health anxiety or catastrophizing interpretations of bodily sensations [4, 8, 9]. The resultant functional impairment extends beyond school absenteeism to include significant disruptions in academic performance, social withdrawal, and increased healthcare utilization due to repeated medical consultations and unnecessary diagnostic procedures [4]. For instance, studies indicate that adolescent SSD patients frequently exhibit comorbid psychiatric conditions (e.g., depression, self-harm behaviors) and high rates of readmission (25% within one year), underscoring the disorder’s chronicity and clinical complexity [9]. Meanwhile, the treatment of adolescent somatic patients still faces challenges. Recently, Geremek et al. [10] found in a survey of a German pediatric and adolescent psychiatry inpatient department that 93.8% of hospitalized patients reported physical symptoms in the past 6 months. In addition, A study of 60 adolescent SSD patients admitted to a tertiary children’s hospital in Australia found that one-quarter of patients were readmitted within one year, 65% of admitted patients sought early interdisciplinary assessment (referral), and 62% of patients developed new mental illnesses, including eating disorders and self-harm behavior [11]. Traditional psychopathological models often conceptualize SSD as a latent construct manifesting through various symptoms. However, an alternative framework—network theory—proposes that mental disorders, including SSD, arise from dynamic interactions among symptoms rather than an underlying disease entity. Within this model, symptoms form a network of mutually reinforcing relationships, where central symptoms may play a critical role in sustaining the disorder by activating other symptoms. Identifying these central symptoms could provide valuable insights for targeted clinical interventions.

Network theory [12] provides a novel framework for understanding SSD, conceptualizing it as a system of dynamically interacting symptoms rather than a latent disease entity. In this model, symptoms form mutually reinforcing networks where central nodes may perpetuate the disorder by activating adjacent symptoms. This approach is particularly suited to SSD given its heterogeneous symptom presentation and the clinical observation that symptom clusters (e.g., nausea, pain, fatigue) often interact in complex, cascading patterns [13]. Furthermore, Bayesian analysis enhances this approach by modeling directional relationships, offering potential causal inferences within symptom networks. While previous studies have explored the symptom networks of SSD in Western populations [13] and Chinese adults [14], research focusing on the symptom network characteristics of Chinese adolescents with SSD remains notably lacking.

This study aims to characterize the network structure of somatic symptoms in adolescent SSD patients in China using network and Bayesian analytical approaches. By identifying central symptoms and their directional relationships, we explore potential clinical implications that may inform future research on network-informed interventions. While these findings require validation through longitudinal and experimental studies, they could contribute to developing more precise clinical strategies for SSD management by investigating how core symptoms may influence the overall symptom network. Specifically, this study addresses several key questions: (1) Which symptoms emerge as central (as indicated by expected influence, EI)? (2) Do networks differ by sex? (3) Which symptoms appear “upstream” in the Directed Acyclic Graph (DAG), suggesting a probabilistic ordering in the network?

## Method

### Participants

This study adopted a cross-sectional design and recruited adolescents from the outpatient department of a tertiary specialized hospital in Shandong Province, China, using convenience sampling between June 2024 to January 2025. Inclusion criteria were as follows: (1) Meeting the DSM-5 diagnostic criteria for SSD. To ensure diagnostic reliability, all patients were independently interviewed and diagnosed by two psychiatrists. The Patient Health Questionnaire-15 (PHQ-15) and the Somatic Symptom Disorder–B Criteria Scale-12 (SSD-12) were further used to evaluate criterion A and criterion B of SSD, respectively. A combined cutoff of PHQ-15 ≥ 9 and SSD-12 ≥ 23 was applied to confirm the diagnosis [15]. (2) Age between 10 and 19 years, in line with the WHO definition of adolescence. (3) Patients and their guardians provided written informed consent after being informed of the study purpose and procedures. Exclusion criteria include language barriers, limited reading skills, cognitive impairment, acute mental illness, or suicidal tendencies. Data quality control: Questionnaires were deemed invalid if they had >20% missing data or logical inconsistencies. Cases with missing data were excluded using listwise deletion, and only complete datasets were included in subsequent analyses. Data were collected electronically via the Wenjuanxing platform, resulting in 1,733 valid questionnaire responses in total. Among them, 585 responses (33.76%) from participants diagnosed with SSD were included in the study. The final sample consisted of 151 males and 434 females, with a mean age of 15.05 years (SD = 1.93). Participants were assured of confidentiality and had the right to withdraw from the study at any time. In addition, demographic and clinical information (e.g., gender, grade, parents’ marital status, socioeconomic status, parenting style, academic performance, and physical activity) were collected to describe the sample.

### Measures

#### Patient health questionnaire-15, PHQ-15

The Chinese version of the 15-item Patient Health Questionnaire (PHQ-15) was employed as the diagnostic A criterion for SSD [16], assessing the severity of common functional somatic symptoms experienced over the preceding 4 weeks. This validated scale evaluates 15 somatic symptoms that collectively encompass over 90% of symptoms encountered in primary care settings [16, 17]. Utilizing a three-point Likert scale, respondents rated symptom intensity as: 0 (not bothered at all), 1 (bothered a little), and 2 (bothered a lot), with higher scores indicating greater symptom severity. This study excluded two adult-specific items (menstruation and sexual activity), resulting in a total of 13 items used [18]. The Chinese version of PHQ-15 has shown good reliability and validity in general population studies [19]. The Cronbach’s coefficient in this study is 0.903.

#### Somatic symptom disorder - B criteria scale, SSD-12

The Somatic Symptom Disorder B Standard Scale (SSD-12) was developed based on the new diagnostic criteria for Somatic Symptom Disorder (SSD) in DSM-5 to evaluate the newly added B standard [20, 21]. SSD-12 measures the psychological burden caused by physical symptoms at the cognitive, emotional, and behavioral levels. This scale consists of 12 items, with scores ranging from 0 (never) to 4 (very frequently) for each item, and a total score ranging from 0 to 48. The reliability and validity of its Chinese version have been fully validated [21]. The Cronbach’s coefficient in this study is 0.938.

### Statistical analysis

Descriptive analyses were conducted using SPSS, while network analysis was performed using R (version 4.4.2). Prior to network analysis, descriptive statistics characterized participant sociodemographics (gender, grade, residence, parenting style, electronic device use; Table 1) and somatic symptom profiles. The 13 PHQ-15 item scores were summarized using mean, SD, skewness, and kurtosis (Table 2). Total scores for the PHQ-15 and SSD-12 were also calculated to characterize overall symptom dimensions (Table 3).

#### Network estimation

Network estimation and visualization were conducted in R (version 4.4.2) using the bootnet and qgraph packages. The 13 somatic symptom items were treated as ordinal variables (scored 0–2). To account for their ordinal nature, a polychoric correlation matrix was first estimated, upon which a Gaussian Graphical Model (GGM) was constructed to represent the partial correlation structure among symptoms. The network was regularized using the Extended Bayesian Information Criterion graphical LASSO (EBICglasso) with a tuning parameter γ = 0.5, which penalizes weaker associations and yields a sparse, interpretable network structure [22]. The resulting edges represent regularized partial correlations between symptoms, controlling for all other nodes in the network.

The expected influence (EI) was used to identify the most central symptoms among the thirteen somatic symptoms, as it captures both positive and negative associations with adjacent nodes, thereby indicating the strength of a node’s direct connections within the network [23]. In addition, we employed strength centrality to examine the direct connections of nodes within the network. Reflecting the sum of all absolute edge weights a node is directly connected to, strength centrality quantifies the connectivity of a node to all other nodes of the network. We use predictability (PRE) to quantify the degree to which the variance of a node can be explained by its directly connected nodes, as an indicator of network controllability. A node with high PRE suggests that its state can be influenced through its neighbors. The predictability (PRE) of each node was estimated using the mgm package in R [24].

#### Estimating network accuracy and stability

The network stability was assessed using the correlation stability coefficient (CS-coefficient) via a case-drop bootstrapping procedure [25]. A CS-coefficient >0.25 indicated moderate node stability, while values exceeding 0.5 reflected strong stability [26]. The network accuracy was evaluated through a non-parametric bootstrapping procedure with 95% confidence intervals (CIs), where narrower intervals denoted higher precision. To statistically compare centrality and edge weights, bootstrapped difference tests were conducted to determine significant variations between nodes and edges.

#### Networktree

To explore whether the network structure of somatic symptoms differed across gender, we applied the networktree algorithm [27, 28] implemented in the R package networktree. A network tree analysis combines network psychometrics with model-based recursive partitioning, a semi-parametric method that recursively tests whether covariates explain heterogeneity in network structure. In our case, the 13 items of the Patient Health Questionnaire (PHQ-15) were specified as node variables, and gender (male vs. female) was entered as the partitioning variable. The algorithm begins with a single network estimated for the full sample using regularized partial correlations based on the graphical LASSO (glasso). It then evaluates parameter instability with respect to the splitting variable. If a significant instability is detected, the sample is split at the optimal cut-point of the covariate, resulting in a tree structure where each terminal node contains a subgroup with its own network model.

In the present study, we specified correlation-based networks with the EBIC glasso estimator as implemented in the qgraph and bootnet packages, which are independent of sample size by definition. To control for spurious splits, we used a conservative significance threshold (α = 0.01) and applied Bonferroni correction across all possible split points.

#### Network models with sex as a covariate

Given the established evidence suggesting potential gender-specific patterns in adolescent somatic symptoms [29], we reconstructed the network model by including gender as a covariate. Specifically, we re-estimated two networks: the original model and a gender-adjusted model. For each network, we calculated the correlation between the edge weight matrices of the original and gender-adjusted models to assess the extent to which accounting for gender influenced the network structure.

Two complementary approaches were used to assess potential sex-related heterogeneity: (1) the networktree algorithm for formal structure comparison, and (2) a covariate-adjusted model including sex.

#### Bayesian network (BN)

BN estimation was performed utilizing the hill-climbing algorithm implemented in the bnlearn package, which generated a directed acyclic graph (DAG) to characterize conditional dependencies [30]. To ensure BN stability, a bootstrap approach with 1000 iterations was used, retaining edges present in at least 85% of iterations with consistent directionality in over 50%. The final averaged network was visualized using the R package Rgraphviz.

## Results

### Sample characteristics

A total of 585 responses were collected. The sociodemographic attributes and other clinical characteristics of the participants are presented in Table 1. The detailed information on the mean, standard deviation, skewness, and kurtosis of physical symptoms based on PHQ-15 is shown in Table 2. According to PHQ-15, the average (SD) total score is 17.4 ± 4.4. The average score for the symptom “fainting” is the lowest, while the average score for “feeling tired” is the highest.

**Table 1:** General information on patients with somatic symptom disorder in adolescents. (*N* = 585)

Variables	*N*	%
**Gender**		
Male	151	25.8
Female	434	74.2
**grade**		
Primary	23	3.9
Junior	290	49.6
Senior	257	43.9
University	15	2.6
**Residence**		
Urban	307	52.5
Town	218	37.3
Rural	60	10.3
**Parenting style**		
Authoritarian	108	18.5
Neglectful	100	17.1
Authoritative	177	30.3
Permissive	35	6.0
Others (Democratic, Encouraging, etc.)	165	28.2
**Personal electronic device usage**		
Never	4	0.7
Occasionally	24	4.1
Sometimes	81	13.8
Frequently	208	35.6
Daily	268	45.8
**Sports**		
Never	278	47.5
Occasionally	181	30.9
Sometimes	69	11.8
Frequently	26	4.4
Daily	31	5.3

**Table 2:** Item description of somatic symptoms as measured by the PHQ-15 (*N* = 585)

	M	SD	Skewness	Kurtosis	%(Absence)	%(Presence)
1.Stomachache	1.41	0.635	-0.611	-0.59	8.0	92.0
2.back pain	1.18	0.776	-0.329	-1.271	22.5	77.5
3.Pain in the arms, legs, or joints	1.19	0.761	-0.337	-1.208	21.1	78.9
4.Headaches	1.63	0.568	-1.258	0.601	4.4	95.6
5.Chest pain	1.13	0.76	-0.228	-1.24	23.0	77.0
6.Dizziness	1.49	0.67	-0.947	-0.287	9.9	90.1
7.Fainting	0.43	0.656	1.257	0.338	66.4	33.6
8.heart is thumping	1.44	0.662	-0.77	-0.503	9.5	90.5
9.Cannot breathe	1.53	0.622	-0.972	-0.102	6.8	93.2
10.Constipation	1.2	0.761	-0.357	-1.198	20.8	79.2
11.Nausea	1.41	0.694	-0.749	-0.637	11.9	88.1
12.Feeling tired	1.84	0.386	-2.206	3.923	0.6	99.4
13.Sleep problems or worries	1.55	0.658	-1.143	0.097	9.2	90.8

**Table 3:** Descriptive statistics of PHQ-15 and SSD-12 dimension scores (*M*, SD). (*N* = 585)

Variables	M	SD
**PHQ-15**		
PHQ Total	17.4	4.4
**SSD-12**		
cognitive	9.8	3.3
affective	12.0	2.9
behavioral	11.4	3.1

### Network analysis

#### Analysis of the structure of the network and the centrality measure

**Fig. 1:**
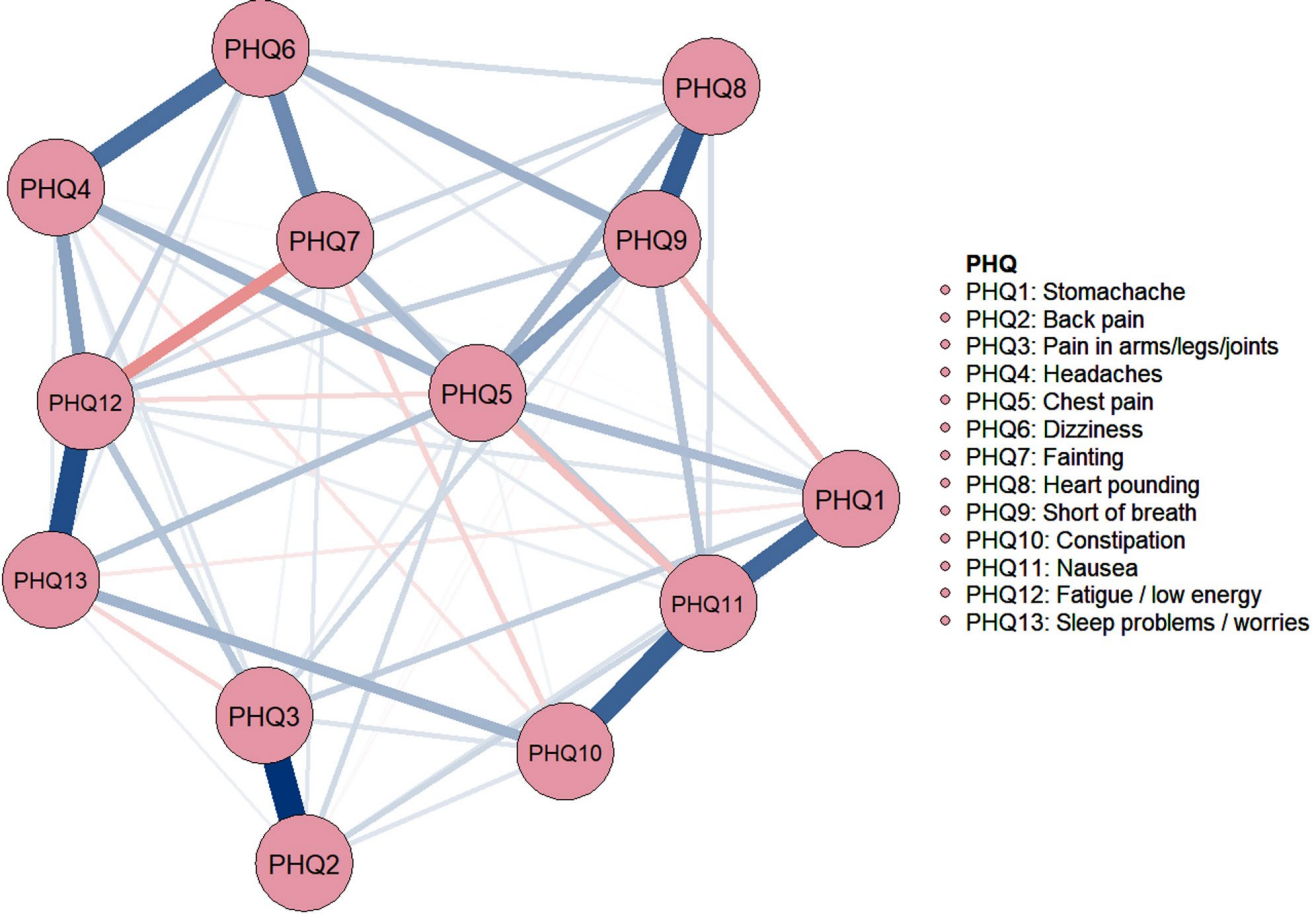
The undirected network structure of 13 somatic symptoms in adolescent patients with somatic symptom disorders. (In the figure, the solid blue line represents positive correlations; the red dashed line represents negative correlations. The edge thickness of the line represents the strength of the association between symptom nodes.)

**Fig. 2:**
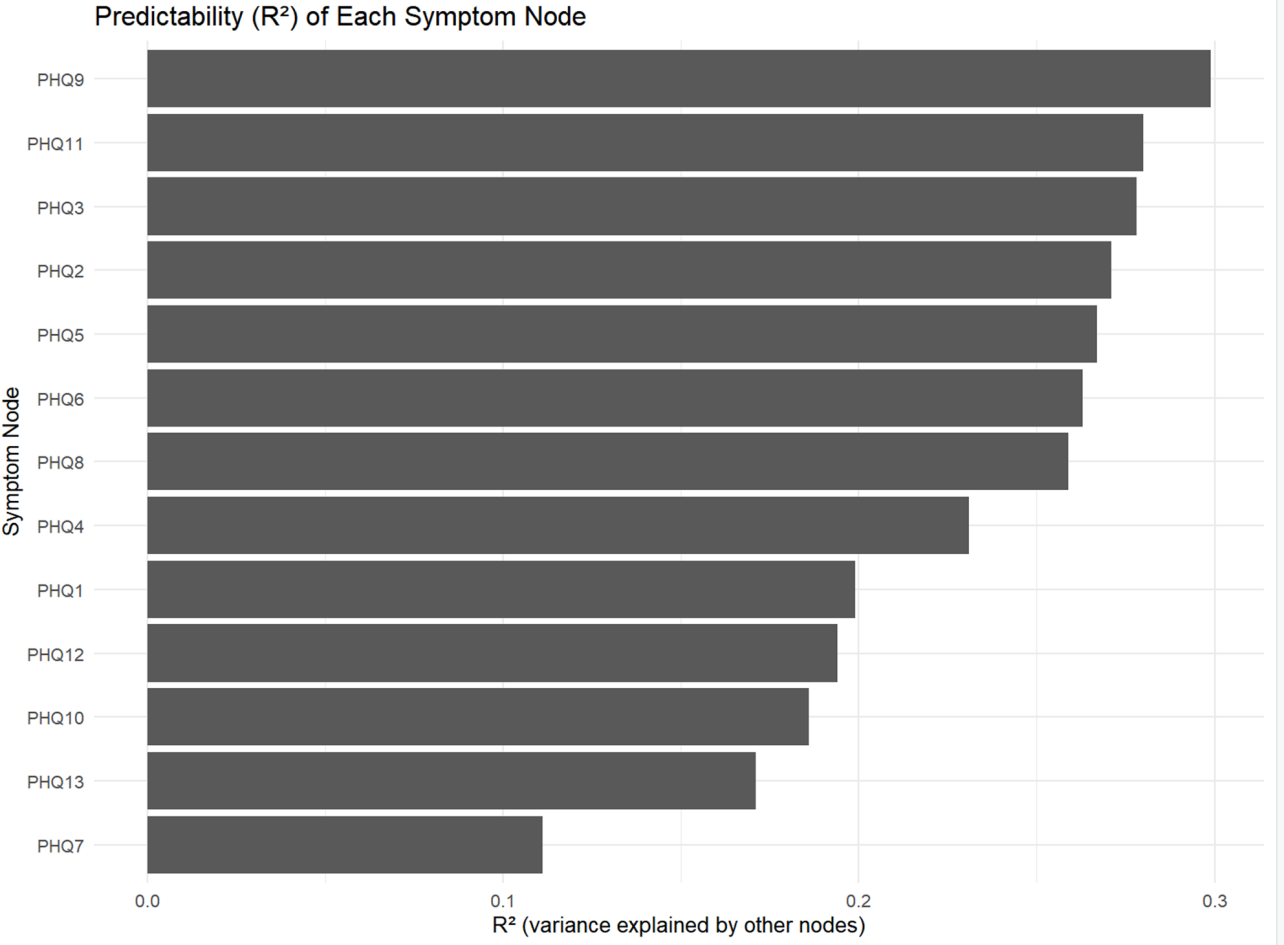
Predictability (R²) of each symptom node in the SSD network. The bar chart displays the proportion of variance (R²) in each symptom explained by all other connected nodes, representing node-level predictability

The network of somatic symptoms with SSD is shown in Fig. 1. The nodes are well-connected, with 59 non-zero edges out of 78 edges (75.6%) and an average weight of 0.068. Among these, 49 edges represent positive partial correlations and 10 represent negative partial correlations. In addition, node predictability visualization is shown in Table S1. Node predictability values (R²) ranged from 0.102 to 0.299, indicating moderate to high levels of variance explained by neighboring nodes (Fig. 2). PHQ9 (‘cannot breathe’) exhibited the highest predictability (R² = 0.299), followed by PHQ11 (‘nausea’), suggesting that these symptoms are more strongly determined by their directly connected nodes within the network (Table S1). Within the SSD patient’s symptom community, node PHQ2 (“back pain”) had the most direct connection with node PHQ3 (“pain in the arms, legs, or joints”), followed by the connections between nodes PHQ12 (“Feeling tired”) and PHQ13 (“Sleep problems or worries”), PHQ8 (“heart is thumping”) and PHQ9 (“cannot breathe”), and PHQ10 (“constipation”) and PHQ11 (“nausea”). Detailed information on edge weights is provided in Table S2.

**Fig. 3:**
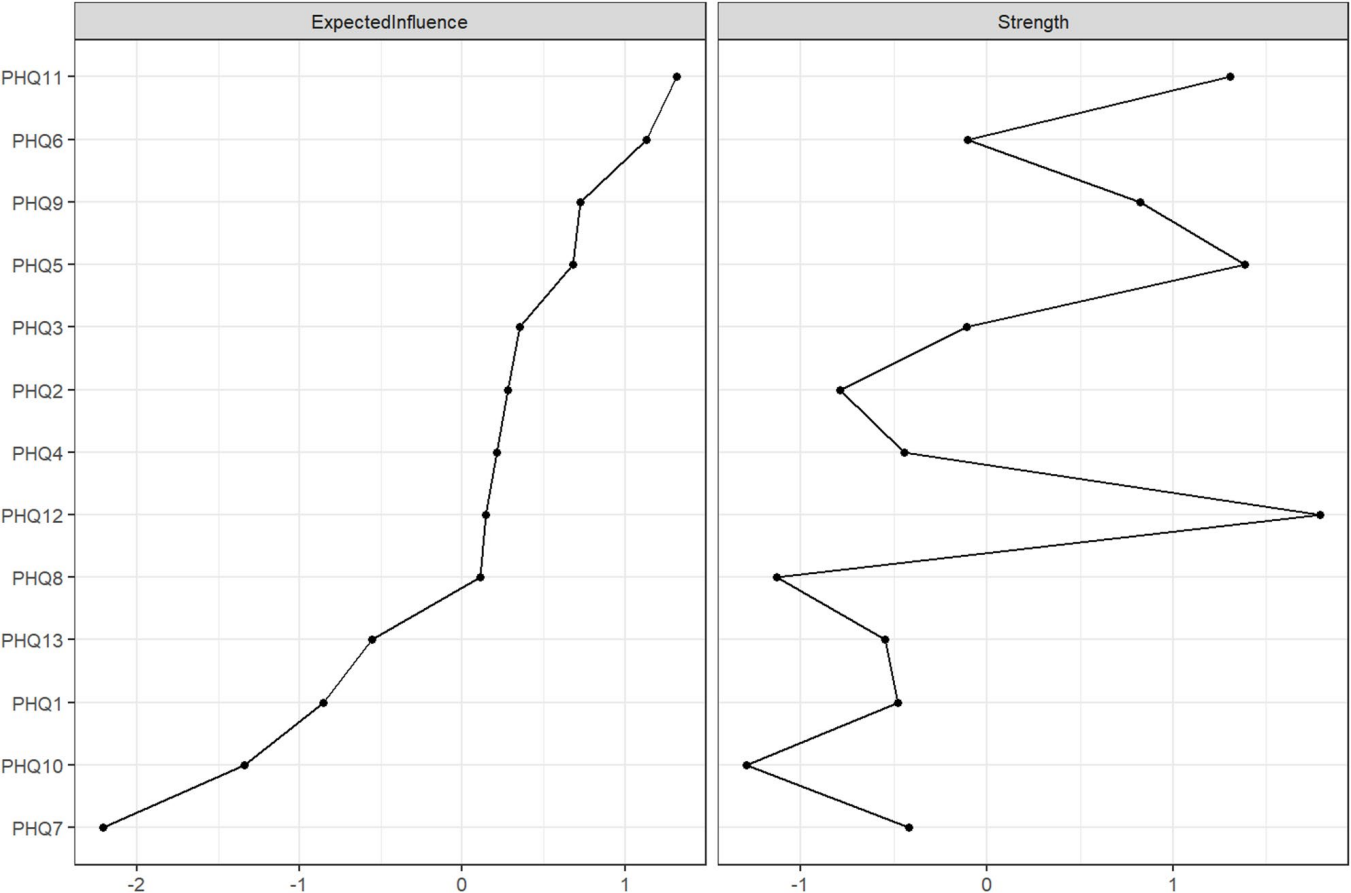
Standardized EI and Strength for each node in the Fig. 1. network

Regarding centrality, nodes PHQ11 (‘nausea’) and PHQ6 (‘dizziness’) showed the highest values of expected influence, while nodes PHQ9 (‘cannot breathe’) and PHQ5 (‘chest pain’) also demonstrated relatively elevated centrality indices compared to most other symptoms (Fig. 3).

#### Network stability and accuracy, and significance testing

The accuracy of the edge weights and centrality indices was supported by the bootstrap procedure, as indicated by relatively narrow 95% confidence intervals (Figure S1). The case-dropping bootstrap approach further showed that the stability of the EI index was robust (CS-coefficient = 0.516), while strength centrality showed lower but acceptable stability (CS-coefficient = 0.128).

To evaluate whether centrality differences were statistically significant, bootstrapped difference tests were conducted (Figures S3 and S4). Figure S2 presents the edge-weight difference test based on nonparametric bootstrapping. Each cell represents the comparison between two edges in the network. Dark-shaded cells indicate statistically significant differences in edge strength, while gray cells indicate non-significant differences. The dense dark areas suggest that several edges differ significantly from most others, implying heterogeneity in edge strengths and highlighting certain symptom pairs as more strongly connected within the network. In particular, the edge between PHQ2 and PHQ3 exhibited a significantly stronger connection compared with most other edges, indicating a robust association between these two somatic symptoms.Taken together with the bootstrapped significance tests, PHQ11 (“nausea”), PHQ-6 (“dizziness”), PHQ-9 (“cannot breathe”), and PHQ-5 (“chest pain”) can still be regarded as comparatively influential nodes within the SSD symptom network, although interpretations should remain cautious.

#### Network tree analysis

The networktree algorithm was applied to examine whether the structure of PHQ-15 symptom networks differed across gender. The analysis did not detect any significant split based on the covariate sex. Specifically, the algorithm resulted in a tree with only a single root node and no further partitioning, indicating that the estimated network structure of somatic symptoms was homogeneous between male and female participants. Thus, no evidence for gender-specific heterogeneity in the PHQ-15 network was observed in the current sample.

#### Network comparison between network models with a covariate

The Spearman correlations between the original and sex-adjusted network models were high across the two networks, indicating minimal structural differences after controlling for gender. Specifically, the correlation coefficients were *r* = 1.000 (*p* < .001) for the overall symptoms network. These findings suggest that including sex as a covariate did not substantially alter the network structure.

Both analyses revealed no significant differences between male and female networks, suggesting that the symptom network structure was stable across sexes.

#### Bayesian network (BN)

**Fig. 4:**
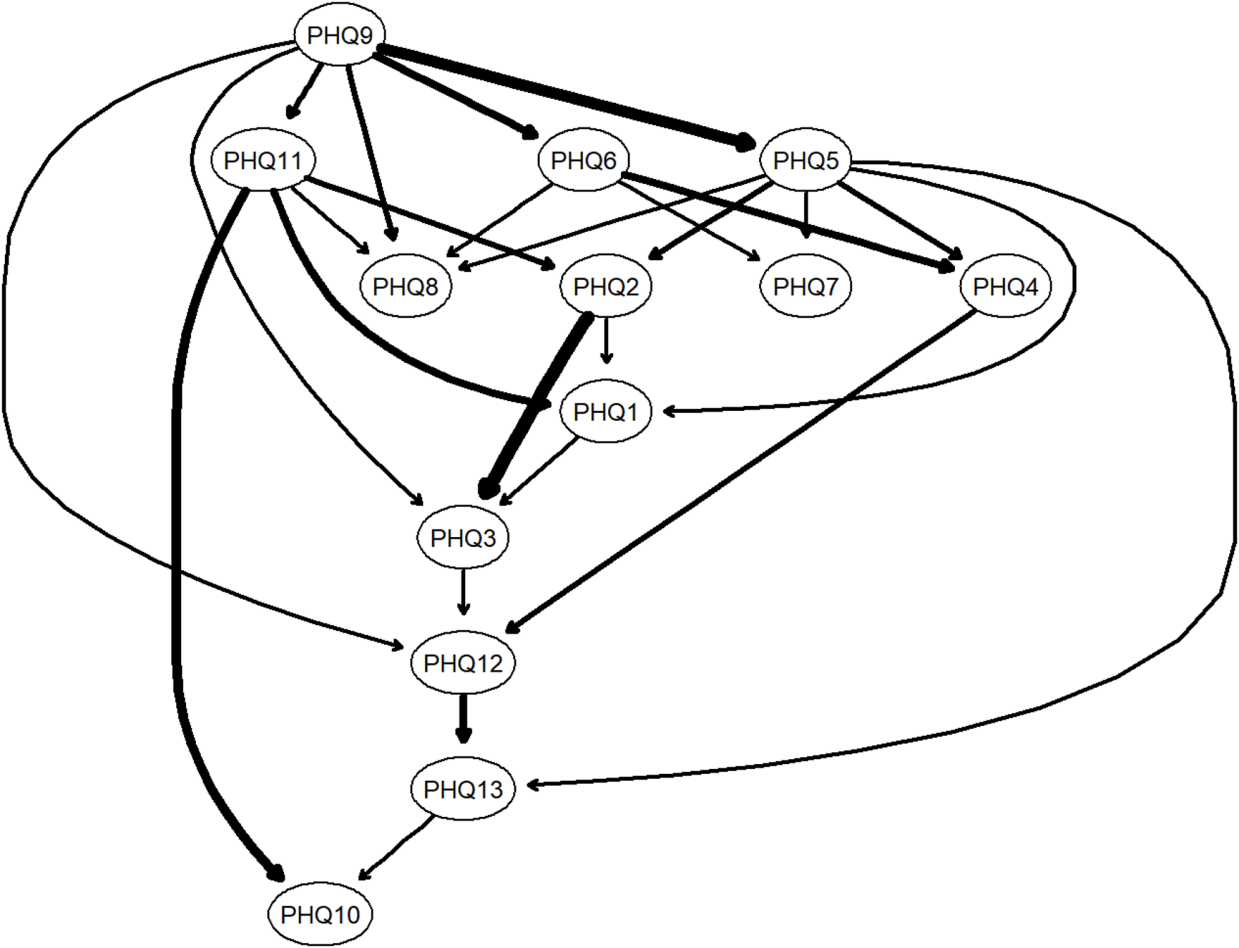
Bayesian network estimated using the hill-climbing algorithm displayed as a Directed Acyclic Graph (DAG)

Figure 4 shows the Bayesian network of the relationship between somatic symptoms in adolescents. PHQ9 (“cannot breathe”) is located at the top of the network, indicating its priority and direct connection with multiple symptoms, which may suggest a potential upstream position within the symptom network. Three other central nodes in the undirected network—PHQ-6, PHQ-5, and PHQ-4—were located in the middle of the DAG, suggesting that they are susceptible to influence from other symptoms while potentially exerting effects on downstream symptoms. The overall BIC score of the network was − 7145.54, reflecting good model fit. Edge stability was further supported by bootstrapped inclusion frequencies, with all retained edges appearing in more than 85% of bootstrap samples and consistent directionality in over 50%.

## Discussion

The present study employed network analysis and Bayesian inference to examine the structure of somatic symptom networks in patients with SSD in a Chinese clinical sample. In addition, we specifically examined whether the network structure of somatic symptoms differed across male and female participants using a network tree approach. The analysis did not detect any significant split by sex, indicating that the network organization of somatic symptoms was homogeneous between genders in this adolescent SSD sample. Importantly, our interpretation focuses on expected influence (EI), which demonstrated adequate stability, whereas strength centrality was unstable and was therefore not used for inference. The analysis revealed that four symptoms emerged as the most central components of the network, characterized by the highest expected influence values. These core symptoms were strategically positioned at the center of the network diagram, suggesting they maintain strong connections with other somatic symptoms and may perpetuate symptom cascades. The DAG further indicated that “cannot breathe” may occupy a predominant position in the network, with potential associations suggesting it could influence or be linked to other somatic complaints. These results align with Yang Xiangyun’s research on 1497 general hospital outpatients, which demonstrated significantly higher cardiothoracic discomfort scores in patients with multiple somatic symptoms [31], while extending previous SSD research by elucidating specific symptom pathways in Chinese patients.

### Central symptoms in SSD and clinical implications

Value beyond latent models. Unlike latent-variable approaches that summarize shared variance, network analysis adds actionable detail: it identifies symptom–symptom pathways (e.g., the PHQ8 “heart is thumping”–PHQ9 “cannot breathe” link) as candidate intervention targets, prioritizes nodes via EI, and reports predictability (R²) as an index of neighbor-driven controllability. Together, these outputs yield clinically testable hypotheses not available from latent models. The network analysis incorporating sex as a covariate, together with the Network Tree approach, revealed no significant sex differences. This finding may be attributable to the limited neuroendocrine and psychosocial sex divergence during adolescence, combined with comparable academic stress and familial expectations among Chinese adolescents, which may help explain the observed similarity in somatic symptom network structures between males and females. The centrality of “nausea”, “dizziness”, “cannot breathe” (chest tightness) and “chest pain” underscores their pivotal role in the SSD symptom network. These reflect two clinically significant patterns: (1) Although the present cross-sectional network analysis cannot adjudicate underlying biological mechanisms, one plausible hypothesis is that these symptoms may be linked to hypothalamus–pituitary–adrenal (HPA) axis hyperreactivity to psychological stress [32]. Within this account, patients could misattribute interoceptive arousal to cardiac pathology, potentially contributing to increased healthcare utilization and health anxiety in Chinese populations [33, 34]. Reflecting this cardiothoracic symptom linkage, our network analysis revealed a strong connection between “heart is thumping” and “cannot breathe,” among the highest-weighted edges in the network. In particular, HPA overactivity could plausibly contribute to sensations such as chest tightness; however, prospective or experimental studies are required to test this mechanism. Clinical observations indicate that patients often misinterpret these manifestations as signs of cardiac disease, leading to excessive healthcare utilization (including repeated cardiology consultations and redundant cardiac examinations) and avoidance of daily activities. Such overutilization of medical services may exacerbate patients’ health anxiety, manifested as excessive focus on physical symptoms and potential triggering of additional symptom clusters [34]. Consequently, in the clinical management of adolescent SSD, special attention should be given to patients’ reports of chest tightness. Establishing early identification mechanisms and precision management protocols may effectively alleviate somatic symptoms and enhance overall treatment outcomes in this population [34, 35]. and (2) From 1990 to 2021, the burden of headache disorders in China has continued to increase. This rising trend has been driven by a combination of factors, including sedentary lifestyles, increasing rates of obesity, elevated psychological stress, more frequent use of electronic devices, and insufficient sleep [36]. Chronic pain, often accompanied by dizziness and headache, triggers significant physical and psychological distress. When combined with social determinants (e.g., economic stress, lack of support) and individual vulnerability factors (e.g., genetic predisposition, maladaptive coping), this interplay may accelerate the development of SSD. Notably, SSD may also serve as both a precursor and perpetuating factor for chronic pain. Emerging evidence suggests that SSD lowers pain tolerance, creating a vicious cycle where heightened somatic awareness amplifies discomfort, further reinforcing dysfunctional illness behaviors [37]. This bidirectional relationship complicates clinical management, as patients often present with overlapping physical and psychological symptoms. This strong association may stem from central sensitization mechanisms leading to hyperalgesia, or reflect catastrophizing cognitive tendencies toward physical discomfort among adolescent patients [38]. Notably, the pair of symptoms-heart is thumping and shortness of breath-showed high co-occurrence, which aligns with typical manifestations of anxiety-related somatic symptoms. Previous studies indicate that such cardiopulmonary symptoms are often triggered by autonomic nervous system dysfunction and are particularly prominent in adolescent populations [39]. Of particular interest is the connection strength between constipation and nausea, which provides new evidence for understanding gut-brain axis dysregulation in SSD patients. Recent neuro-gastroenterological research suggests this association may reflect abnormal interactions between the enteric nervous system and central nervous system [40].

### Probabilistic ordering of cardiothoracic symptoms: a bayesian perspective

Importantly, “cannot breathe” demonstrated the highest predictability in the undirected network analysis, and the directed acyclic graph (DAG) further placed this symptom at the apex of the probabilistic causal hierarchy, suggesting that it may serve as a key driver within the somatic symptom network. Dysfunctional breathing has been clinically observed in patients with a range of functional somatic disorders, including irritable bowel syndrome (IBS), chronic fatigue syndrome (CFS) [41, 42]. Existing evidence suggests that disordered breathing may play a significant role as a predisposing, precipitating, and perpetuating factor in persistent somatic symptoms [43, 44]. Notably, the symptom of “cannot breathe” identified in this study may be closely associated with dysregulation of the autonomic nervous system (ANS) [45]. Individuals with reduced parasympathetic activity typically exhibit lower respiratory sinus arrhythmia (RSA) and diminished variability in both respiratory and heart rates, reflecting poor autonomic flexibility [46]. Respiration, uniquely regulated by both conscious and unconscious control, is highly sensitive to emotional and environmental stressors, with stress often inducing shallow, rapid hyperventilation [47]. This breathing pattern results in hypocapnia, which is associated with symptoms such as dizziness, tingling sensations, and heightened anxiety. Such abnormalities in breathing not only exacerbate distress but may also alter central nervous system (CNS) functioning, contributing to the emergence of somatic symptoms like chest pain, chest tightness, and dyspnea. These findings may help explain why disordered breathing symptoms, particularly the experience of “cannot breathe”, serve as upstream nodes in adolescent symptom networks, potentially amplifying other somatic symptoms.

### Limitations

Several limitations should be acknowledged. First, the cross-sectional design precludes definitive causal conclusions; longitudinal or experimental network studies are needed to track symptom dynamics and test directional hypotheses over time. Second, the sample was recruited by convenience sampling from a single tertiary hospital in China, which may limit generalizability to other cultural and healthcare contexts; the findings may not directly extend to Western populations. Third, although data collection was conducted electronically, online self-report can introduce selection and measurement biases (e.g., differential access/engagement, recall bias, and common-method variance), which should be considered when interpreting effect sizes and network structure.Fourth, while we examined sex-related heterogeneity using a network tree and a sex-adjusted network (which did not reveal meaningful differences), we did not perform formal multi-group comparisons (e.g., NCT) by sex or age groups. The sex imbalance in our sample further limits the precision of subgroup inferences; future studies with balanced samples should implement multi-group (sex/age) network analyses.Finally, with respect to centrality metrics, expected influence (EI) demonstrated adequate stability and was therefore used for inference, whereas strength centrality was unstable (low CS-coefficient) and was not interpreted. Although the Bayesian DAG offers a probabilistic ordering of symptoms, it should be considered hypothesis-generating; experimental or longitudinal data are required to validate any putative upstream–downstream relations.

### Conclusion

This study underscores the utility of network approaches in unraveling the complex interplay of SSD symptoms. By identifying nausea, dizziness, cannot breathe, and chest pain as central and potentially causal nodes, our findings highlight priority targets for clinical intervention. Future treatment research should explore whether personalized interventions focusing on these key symptoms yield superior outcomes compared to conventional approaches. Ultimately, integrating network-based strategies into SSD care could pave the way for more precise and effective treatments.

## Supplementary Information

Below is the link to the electronic supplementary material.

Supplementary Material 1

## Data Availability

Data in the current study is available from the corresponding author on reasonable request.

## Abbreviations

CI: Confidence interval
SSD: Somatic symptom disorder
GGM: Gaussian Graphical Model
BN: Bayesian network
EI: Expected influence
BIC: Bayesian Information Criterion
