# Supplementary material for: Network analysis in adolescent patients with somatic symptom disorders
Source: BMC Psychiatry. 2025 Dec 27;26:96. doi: 10.1186/s12888-025-07686-3 (PMC12853573; doi:10.1186/s12888-025-07686-3)

**Supplemental materials**

**Figure legends**

**Figure S1.** Bootstrapped test for confidence intervals of edge,EI and Strength of somatic symptoms network model

**Figure S2.** Bootstrapped difference test for edge weight of network model

**Figure S3.** Bootstrapped difference test for EI of network model

**Table S1.** Basic information and network inference of 13 somatic symptoms items

|  | EI | Strength | R2 |
| --- | --- | --- | --- |
| PHQ1 | -0.856 | -0.478 | 0.199 |
| PHQ2 | 0.278 | -0.788 | 0.270 |
| PHQ3 | 0.350 | -0.107 | 0.272 |
| PHQ4 | 0.214 | -0.443 | 0.233 |
| PHQ5 | 0.678 | 1.386 | 0.267 |
| PHQ6 | 1.132 | -0.102 | 0.264 |
| PHQ7 | -2.208 | -0.419 | 0.112 |
| PHQ8 | 0.113 | -1.129 | 0.259 |
| PHQ9 | 0.728 | 0.822 | 0.299 |
| PHQ10 | -1.340 | -1.293 | 0.181 |
| PHQ11 | 1.320 | 1.307 | 0.280 |
| PHQ12 | 0.144 | 1.790 | 0.194 |
| PHQ13 | -0.553 | -0.547 | 0.172 |

**Table S2.** Weighted adjacency matrix of somatic symptoms network model

|  | **PHQ1** | **PHQ2** | **PHQ3** | **PHQ4** | **PHQ5** | **PHQ6** | **PHQ7** | **PHQ8** | **PHQ9** | **PHQ10** | **PHQ11** | **PHQ12** | **PHQ13** |
| --- | --- | --- | --- | --- | --- | --- | --- | --- | --- | --- | --- | --- | --- |
| **PHQ1** | 0.000 |  |  |  |  |  |  |  |  |  |  |  |  |
| **PHQ2** | 0.063 | 0.000 |  |  |  |  |  |  |  |  |  |  |  |
| **PHQ3** | 0.107 | 0.454 | 0.000 |  |  |  |  |  |  |  |  |  |  |
| **PHQ4** | 0.025 | 0.039 | 0.057 | 0.000 |  |  |  |  |  |  |  |  |  |
| **PHQ5** | 0.148 | 0.083 | 0.041 | 0.167 | 0.000 |  |  |  |  |  |  |  |  |
| **PHQ6** | 0.039 | 0.000 | 0.000 | 0.322 | 0.000 | 0.000 |  |  |  |  |  |  |  |
| **PHQ7** | 0.003 | 0.055 | 0.025 | -0.008 | 0.147 | 0.260 | 0.000 |  |  |  |  |  |  |
| **PHQ8** | 0.000 | 0.011 | 0.000 | 0.000 | 0.155 | 0.077 | 0.087 | 0.000 |  |  |  |  |  |
| **PHQ9** | -0.107 | -0.016 | 0.083 | 0.000 | 0.224 | 0.175 | 0.000 | 0.361 | 0.000 |  |  |  |  |
| **PHQ10** | 0.033 | 0.057 | 0.061 | -0.046 | 0.023 | 0.000 | -0.074 | 0.000 | 0.000 | 0.000 |  |  |  |
| **PHQ11** | 0.336 | 0.090 | 0.000 | 0.046 | -0.112 | 0.000 | 0.112 | 0.075 | 0.129 | 0.350 | 0.000 |  |  |
| **PHQ12** | 0.057 | 0.000 | 0.127 | 0.210 | -0.067 | 0.105 | -0.199 | 0.073 | 0.105 | 0.000 | 0.039 | 0.000 |  |
| **PHQ13** | -0.043 | 0.035 | -0.072 | 0.047 | 0.135 | 0.052 | 0.000 | 0.000 | 0.000 | 0.167 | 0.000 | 0.397 | 0.000 |

**Figure S1.** Bootstrapped test for confidence intervals of edge of somatic symptoms network model **in the Figure 1. network.**


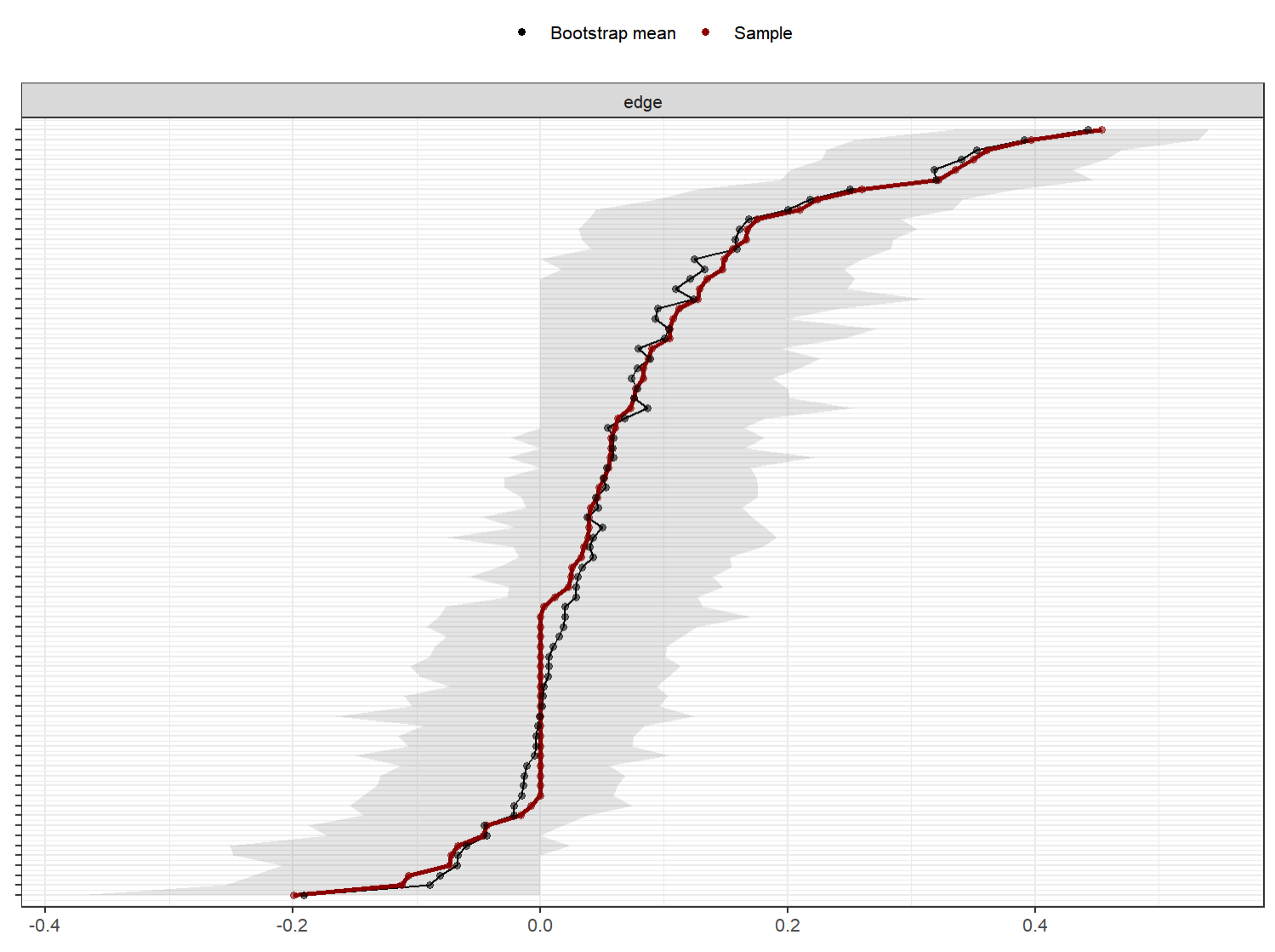


**Figure S2. Strength and EI stability tests in the Figure 1. network..**


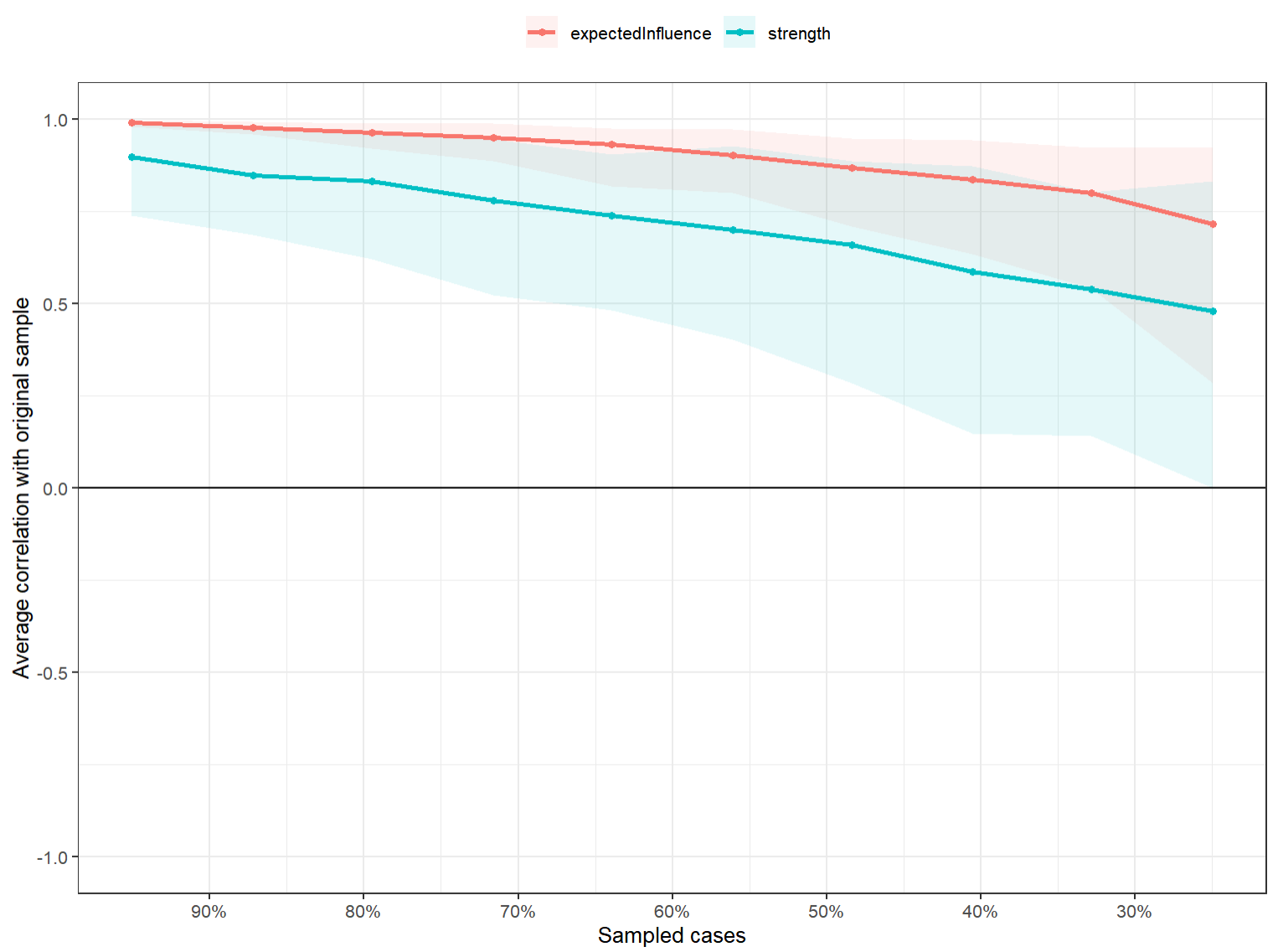


**Figure S3.** Bootstrapped difference test for edge weight of network model **in the Figure 1. network.**


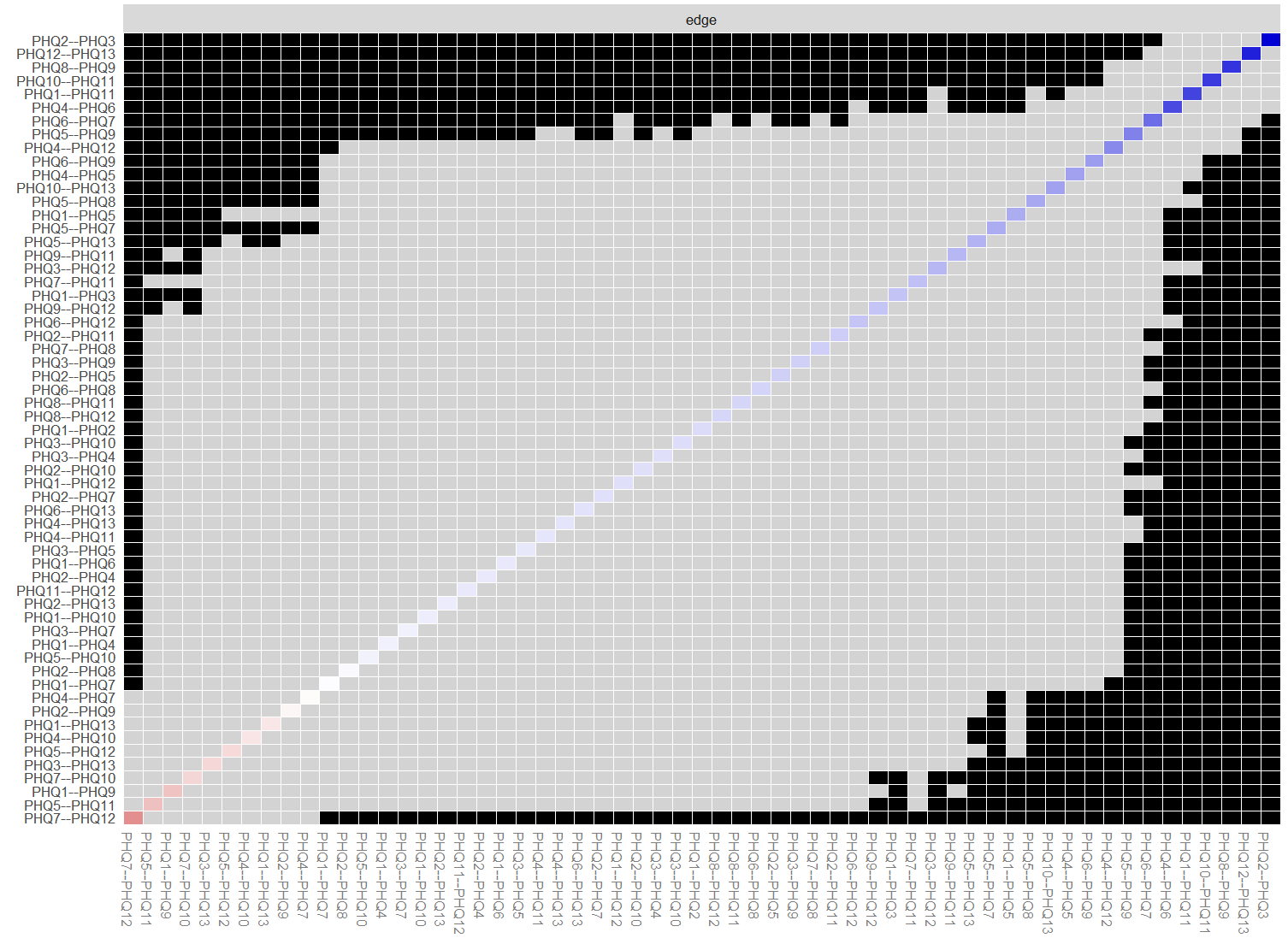


**Figure S4.** Bootstrapped difference test for EI of network model **in the Figure 1. network.**


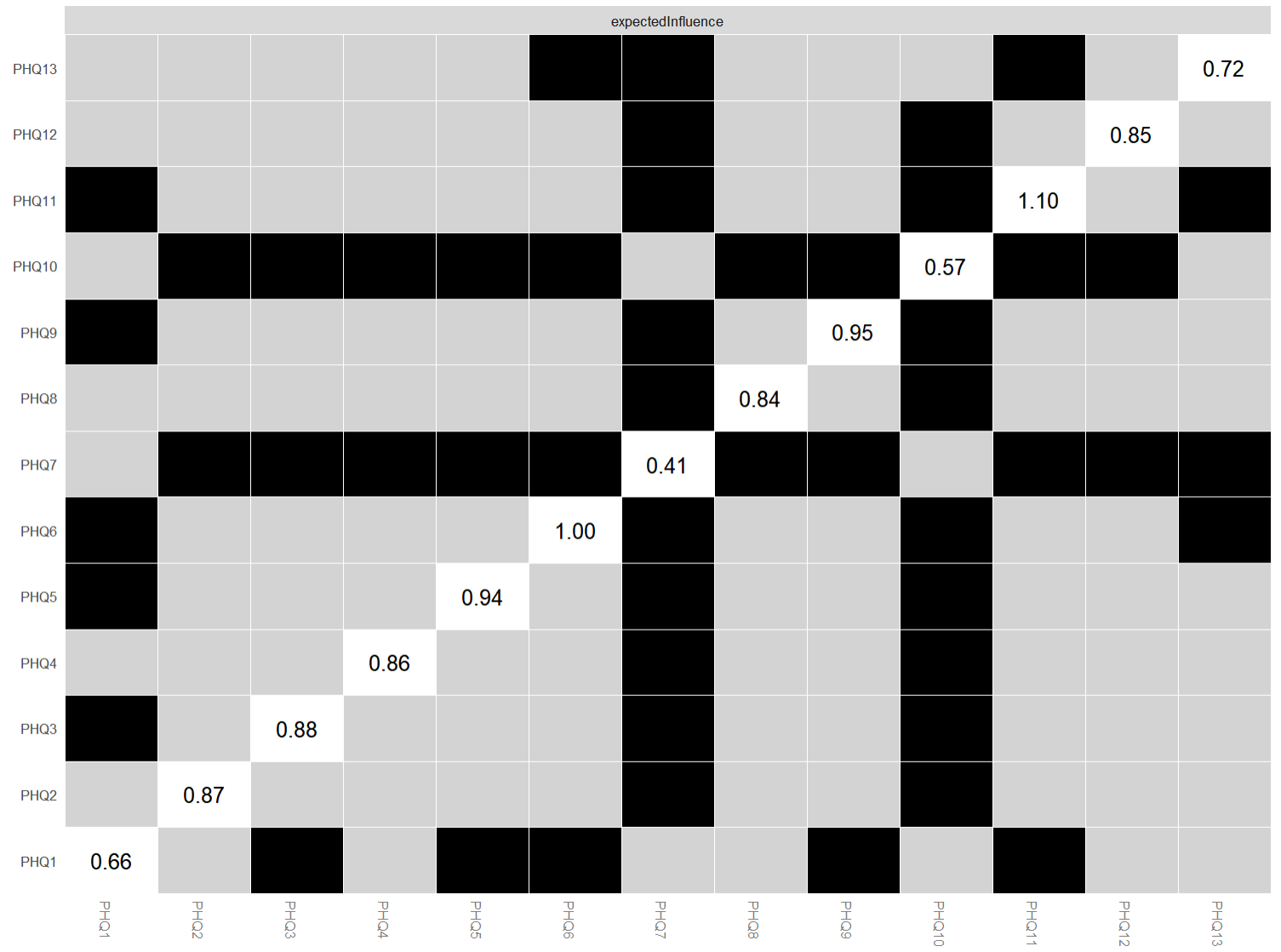

Supplement: Supplementary file 1 — Supplementary Material 1 [file 12888_2025_7686_MOESM1_ESM.docx]
